# Linking Criminal Justice-Involved Individuals to HIV Preexposure Prophylaxis: A Qualitative Analysis of Multiple Stakeholder Perspectives

**DOI:** 10.1177/23259582251341940

**Published:** 2025-05-11

**Authors:** Jessica Lee, Robin T. Higashi, Timothy P. Hogan, Julia L. Marcus, Emily C. Repasky, M. Brynn Torres, Douglas Krakower, Ank E. Nijhawan

**Affiliations:** 1 12334 UT Southwestern Medical Center, Department of Medicine, Dallas, TX, USA; 2 12334 UT Southwestern Medical Center, O’Donnell School of Public Health, Dallas, TX, USA; 3 Center for Health Optimization and Implementation Research (CHOIR), 581361 VA Bedford Healthcare System, Bedford, MA, USA; 4 Department of Population Medicine, Harvard Pilgrim Healthcare Institute, Boston, MA, USA; 5 Division of Infectious Diseases, 1859 Beth Israel Deaconess Medical Center, Boston, MA, USA

**Keywords:** HIV prevention, incarcerated populations, stigma, linkage to care, correctional health, HIV education

## Abstract

**Objective:**

Although incarcerated individuals are at disproportionately higher HIV risk compared to the general US population, few jails offer linkage to preexposure prophylaxis (PrEP). We explored stakeholder perspectives about barriers and facilitators to PrEP for justice-involved individuals.

**Methods:**

Semi-structured interviews were conducted with three stakeholder groups in Dallas County, Texas: justice-involved individuals (*n* = 8), County Jail staff (*n* = 9), and employees of local community organizations that provide PrEP services (*n* = 9). Transcripts were analyzed using a combined deductive and inductive approach.

**Results:**

Barriers to PrEP linkage included: limited provider knowledge of and capacity for PrEP care, stigma around incarceration and PrEP, and mistrust in healthcare and criminal justice systems among justice-involved individuals. Perceived facilitators included addressing competing priorities, partnering with community organizations, and providers' cultural competency training.

**Conclusion:**

Future research should focus on adapting successful implementation strategies to the needs of justice-involved populations to improve HIV prevention and health outcomes in high-burden regions like the Southern USA.

## Introduction

The prevalence of HIV diagnoses among incarcerated populations in the USA is five times that of the non-incarcerated adult population.^1^ Incarcerated populations experience high rates of HIV infection due to multiple overlapping risk factors such as transactional sex, low condom use, high incidence of sexually transmitted infections (STIs), dissolution of primary intimate partnerships, and socioeconomic barriers to medical care such as unemployment and homelessness.^2,3^ In 2006, among the 1.2 million people with HIV (PWH) in the USA, 168,000 were released from jails and prisons, accounting for around 14% of PWH.^4^

Populations at increased risk of HIV are overrepresented in jails and prisons in the Southern USA. The National Inmate Survey, a nationally representative survey of 106,532 incarcerated people, found that men who have sex with men (MSM) were disproportionately represented, with over 94,000 MSM in the US criminal justice system.^5^ MSM, in particular those in the South, are disproportionately affected by HIV: in 2022, 68% of new HIV diagnoses in Dallas County were among MSM.^6^ In addition, Black and Latinx populations are overrepresented in prison and jail populations and experience higher rates of HIV infection in the Southern USA.^7,8^ Of new infections in Dallas County in 2022, 47% and 35% were among Black and Latinx individuals, respectively.^6^ Dallas County also had an HIV incidence rate of 42 cases per 100,000 population in 2022,^6^ ranking first among all counties in Texas.

Given these statistics, the criminal justice system in the South represents an opportune setting for HIV prevention.^9-11^ Preexposure prophylaxis (PrEP) can decrease new HIV infections by 99%;^12,13^ however, virtually no jails across the US offer linkage to PrEP at community re-entry due to barriers at multiple ecological levels, including those that are individual (e.g., low perceived risk and PrEP knowledge among potential consumers), dyadic (e.g., limited patient-provider communication about PrEP in jails), and structural (e.g., lack of access to sexual health clinics upon release).^14-18^ A program to initiate PrEP in the correctional system and provide linkage to PrEP care for individuals upon reentry was implemented in Rhode Island in 2019; however, only about 15% of individuals received medication delivery, and less than 3% of individuals were successfully linked to PrEP care after release.^19^

Prior research on ways to address the underuse of PrEP for justice-involved individuals in the Southern USA has been limited in scope.^20-23^ Qualitative studies in Rhode Island and Arkansas have explored barriers to linkage to PrEP care through the perspectives of high-risk incarcerated individuals including MSM, women, and injection drug users, as well as stakeholders including administrators, health care providers, and social workers.^21,24,25^ However, the perspectives of other key stakeholders, such as security staff in jails, public health leaders, or community organizations have not yet been garnered. Gaining a more comprehensive understanding of varied stakeholder perspectives is crucial to developing effective, community-engaged strategies for linking individuals to PrEP as they are released from the criminal justice system.

Our objective in this study was to explore multiple stakeholders’ perspectives on perceived barriers and facilitators to PrEP linkage to care among individuals involved with the criminal justice system.

## Methods

### Design and Setting

This study reports formative qualitative work conducted as part of the initial phase of a hybrid implementation-effectiveness trial to develop and test a multi-component implementation strategy for linking justice-involved individuals to PrEP services. We pursued our work in the city of Dallas, Texas, which has been identified by the Centers for Disease Control and Prevention as a “hotspot” for HIV infections.^26^

### Recruitment and Sampling

We selected three stakeholder groups to participate in semi-structured interviews: (1) justice-involved individuals, (2) Dallas County Jail staff, and (3) employees of community organizations that serve individuals accessing PrEP services in Dallas. We recruited justice-involved individuals primarily by attending group meetings held by the HIV/AIDS Re-entry Coalition (HARC) and parole orientation. At these meetings, the opportunity to participate in interviews was announced and the research team contact information was distributed; interested participants then contacted the research team for scheduling. Eligible individuals included those who spoke English or Spanish and who had been incarcerated at the Dallas County Jail. To obtain diverse perspectives from jail staff, we recruited a purposive sample comprising various roles including medical providers, clinical staff (e.g., nurses conducting intake assessments and health educators), and administrative staff (e.g., unit managers and non-clinical staff). Among community organization employees, individuals such as medical providers, case managers, and other social services staff with whom the study team already had existing relationships were invited; the invitation to participate was also extended to other members of their organization whom they felt may be best positioned to discuss a list of interview topics relating to PrEP that was provided.

### Instrument Development

Interview guide questions were rooted in the EPIS implementation science framework^27^ and our identification of gaps in the peer-reviewed literature. Example questions by domain and participant group are listed in Table 1.

**Table 1.** Example Interview Guide Questions by Participant Group and Domain.

Interview guide domain	Participant group	Example questions
Knowledge, experiences with PrEP (EPIS: exploration)	Justice-involved individuals	Please tell me what you know about PrEP. Has any health professional ever talked with you about PrEP before? Please tell me what you remember about that conversation.
	Jail staff	Please tell me what you know about HIV testing and prevention services in the jail. Do you think it's important for the jail to provide HIV prevention and/or sexual health education to people who are incarcerated?
	Community organization staff	Tell me about the PrEP services that your clinic/institution provides. Do you work with justice-involved individuals?
Barriers and facilitators (EPIS: preparation)	Justice-involved individuals	How should jail personnel approach and talk with people who are incarcerated about HIV risk and PrEP? What are the challenges we should anticipate?
	Jail staff	What is the best time or location for providers or health educators at the jail to have discussions with patients about sensitive topics? What seems to work well?
	Community organization staff	What are the resources you need as a community-based provider to be able to link justice-involved individuals to PrEP services and care?
Linkage to care strategy (EPIS: implementation)	Justice-involved individuals	Where would you suggest we approach people about PrEP when they are in jail?
	Jail staff	What are the cultural norms and attitudes of people who are incarcerated that are important for us to consider in planning a linkage to care program?
	Community organization staff	What would help your organization reach justice-involved individuals who need HIV prevention services and PrEP?

### Data Collection

Three study members [RTH, MBT, JL] trained in qualitative methods conducted the semi-structured interviews between October 2022 and May 2023, either in person, via videoconferencing, or via telephone, based on participant preference. All interviews were audio-recorded. After each interview, study members also developed fieldnotes and shared them with the larger research team in weekly team meetings. This process promoted discussion about nuances and variations in participants’ responses to various questions, facilitated interpretation of findings from multiple team members’ perspectives, and identified when thematic saturation had been reached.^28^

Verbal informed consent was obtained for interview participation and audio-recording in accordance with the approved protocol of the Institutional Review Board at UT Southwestern Medical Center and Parkland Health. All audio recordings were transcribed verbatim and de-identified by a professional vendor, and transcript data were managed in NVivo (Lumivero) for analysis.

### Data Analysis

The lead qualitative investigator [RTH] drafted an initial qualitative codebook based on a combined deductive and inductive approach. We supplemented an initial draft consisting of the interview guide domains with emergent findings that were recorded in fieldnotes and discussed in weekly team meetings. Three data analysts [MBT, JL, ER] open-coded three transcripts (10% of total sample, one from each stakeholder group) to pilot the initial codebook and iterate changes as needed. Then the analysts jointly coded three additional transcripts (another 10% of total sample, one from each stakeholder group) to confirm the themes and finalize the codebook. Thereafter, all remaining transcripts (remaining 80% of total sample) were double-coded with discrepancies resolved through discussion. Findings were discussed with stakeholders from the jail and community organizations at subsequent meetings, all of whom found the analysis to be credible.

## Results

Interviews were completed with 26 individuals: eight justice-involved individuals, nine jail staff, and nine employees of community organizations (one of whom had a history of justice involvement). Participant demographic characteristics are shown in Table 2.

**Table 2.** Demographic Characteristics of Interview Participants (*N* = 26).

	Justice-involved individuals (*n* = 8)	Community organization staff (*n* = 9)^a^	County Jail personnel (*n* = 9)
Gender			
Female	1	8	5
Male	7	1	3
Prefer not to respond	0	0	1
Race/ethnicity			
Asian	0	3	3
Hispanic	2	1	1
Non-Hispanic Black	4	3	2
Non-Hispanic White	2	2	2
Prefer not to respond	0	0	1
Age			
< 40 years	2	3	3
40–49 years	2	4	2
50–59 years	2	1	2
≥ 60 years	1	0	0
Prefer not to respond	1	1	2
Professional role			
Clinical provider	0	4	3
Clinical staff	0	0	2
Administrative manager	0	0	3
Administrative staff	0	0	1
Social services staff	0	5	0
Unknown	8	0	0

a One individual (female, White, age 40–49) was both a justice-involved individual and community organization staff.

Several themes emerged from the data, and these have been collated along with illustrative quotations in tabular form (Table 3). Among barriers to PrEP linkage to care, we found: lack of capacity for PrEP and HIV counseling among jail providers due to a significant burden of healthcare needs; limited knowledge of PrEP among jail providers and justice-involved individuals; stigma related to incarceration, sexual and gender minorities, PrEP use, and HIV among jail providers and staff, and justice-involved individuals; and preexisting mistrust in the healthcare and criminal justice system among justice-involved individuals. Among the perceived facilitators identified and described by the three stakeholder groups, we identified: enhancing engagement of justice-involved individuals through digital media in jails; addressing competing priorities for justice-involved individuals to make room for medical care; partnering with community organizations that provide additional social services; overcoming mistrust and stigma; having relatable providers; and communicating candidly about PrEP and HIV.

**Table 3.** Findings by Theme and Exemplar Quotes.

Theme	Quote
*1. Barriers to linkage to PrEP care*	
a) Lack of capacity for PrEP and HIV counseling among medical providers	Honestly, [as a] doctor we cannot discuss everything for hours. I'm telling you there is a batch behind those people. If I sit with one person and take 15 min, my god, it's intolerable. (county jail personnel; health service provider)
	If somebody comes with STD positive, I always discuss sex education with that patient. Otherwise, if somebody thinks that I can do it with everybody, it's not possible. (county jail personnel; health service provider)
	I feel like if you want [a linkage to care program] to be effective, you're gonna want your own staff to do [it]. Because honestly, everyone here is pretty stretched… If the existing staff does it, they might just do it really quickly. And it's not [going to] be effective. (county jail personnel; administrative role)
	I'm doing this interview to explain to you why I don't think providers really can play a role in this. I think the study needs to hire other people to do this… I feel like already with [HIV/STI] labs, that shouldn't be my job but it was just dumped on us and part of our evaluation; all of that was a surprise. So, we're already dealing with that. On top of all the real nuts and [bolts] of medicine that we should be dealing with. (county jail personnel; health service provider)
b) Limited knowledge of PrEP among jail providers and justice-involved individuals	I've had encounters with providers where they tell me that they are not an STI doctor; they are a chronic care provider, or they specifically want to look at bones. If they are not willing to provide care for every situation that may be going on during their visit, it's not gonna be helpful. (county jail personnel; health service provider)
	Who would you tell them to go to for PrEP? We don't even know that… I wouldn't know who to send them to for PrEP right now. (county jail personnel; health service provider)
	All I know about PrEP is what I see up on the commercials and I know that it's for like HIV use or people that are exposed to it or may be exposed to it. (justice-involved individual)
	Oftentimes I feel like patients’ perceived risk is lower than what their actual risk might be for HIV. If you don't actually have a disease that you're treating… to have a patient realize how beneficial it might be for them to come… for labs every three months. They have to come pick up their prescription every month. They have to fill out all this paperwork if they need patient assistance. And so, I think for a preventative, sometimes that might not seem worth it to a patient. (community organization staff; health service provider)
c) Stigma related to incarceration, gender and sexual minorities, PrEP use, and HIV status	Any kind of negative feeling about the test or anything like that will probably cause barriers to patients being tested, because if I don't think the test is right or I don't think you should be asked to test, I'm probably not even gonna try to tell you in a good way, “Hey, this is free and this is an opportunity for you.” If I don't script it in the appropriate way, people are going to turn it down. And I really felt that that was an issue. (community organization staff; administrative role)
	Sometimes people might not know enough about PrEP so they think anybody that takes it or is interested in taking it might be HIV positive or might be homosexual or something like that. (justice-involved individual)
	It's a stigma when you're in jail about stuff like that. And you really don't want nobody talking to you about it at all, especially if you're not involved in that type of behavior. (justice-involved individual)
	There's some smart people that's in jail, there's some really intelligent people in jail, but then there's a lot more small-minded people in jail that create stories and enhance stories about stuff, which could turn into a violent situation because you're taking PrEP – you don't have it, but you're taking it. But someone can turn it up, “Oh, if you're taking it, you got [HIV], and we don't want you in this tank.” (justice-involved individual)
	So then when you come and you offer a blood draw and you're like, “Hey, I'm gonna perform an HIV test,” and it's like, “Whoa, are you saying I have HIV?” And then it's a whole other thing, and they still don't fully understand what HIV is or how it's contracted and then it's like, “Oh, are you calling me gay?” and turns into something else. (county jail personnel; health service provider)
d) Preexisting mistrust in the healthcare and criminal justice system among justice-involved individuals	They don't give us good contact information because they're afraid the sheriff will show up at their place and arrest them again. So, it makes it really difficult to do follow-ups because there's that aspect of fear that any information they give may be used against them in the future. (county jail personnel; administrative role)
	I always take a social history like smoking, drug, alcohol. Some people think medical will cross over to their case, so they don't always wanna share that lifestyle. But if, say for instance, a person here on drug charges or they're using drugs or caught with drugs, they're worried that if they tell me they use drugs it'll be used in their case. (county jail personnel; health service provider)
*2. Facilitators and strategies to linkage to PrEP and HIV care*	
a) Enhancing engagement of justice-involved individuals in the jail setting through digital media	As far as I know, most of the education comes from providers. The issue with that is, if an inmate doesn't have any health issues, they may not ever see a provider while they're here. So, they don't get that extra education, which is where the podcast came in to educate everybody. It's accessible to everybody and they can listen to it whenever they want to replay episodes, go back to whatever it is. (county jail personnel; health service provider)
	I was surprised by the initial response from the inmates and how engaged they seemed to be. I didn't get to do that podcast for that week, and I came back and checked the little questions where they submitted it and they were like, “Where's the podcast? When are you gonna submit them?” And I was like, “All right, I guess it's working, this is great.” So, I'm impressed. (county jail personnel; health service provider)
	I had somebody send me question requests, and they were saying, “Oh, I watched your podcast. I didn't know you could get it like this. How do I get tested, submitted kind of for testing, please?” And they'll come test you any time. So, I think it's really just education for this group while they're here. Once they're released, it opens up a whole other can of worms, like lack of transportation, resources, financial barriers once they're out there. While they're here it's just education, because they can get tested for free. (county jail personnel; health service provider)
	It's gotten to the point where some of the officers will even be like, “Hey, Texas got the highest number,” because I've said it so many times in front of them and they'll even help me to get people to want to test more. I don't think people understand that it's not just men who have sex with men who catch HIV. I don't think that they understand that. I get a lot of [uneducated] people with regards to the information about how you can catch that disease. (county jail personnel; health service provider)
b) Addressing competing priorities for justice-involved individuals to make room for medical care upon community reentry	While at the end of the day, the ultimate goal for our program is linkage to care, that might not necessarily be their ultimate goal. So how do we help everybody get what they need? Whether or not that's linkage to care, whether or not they need some clothes – if you need to do X, Y, Z before medical care, how can we help get that taken care of so that it's easier for you to get linked to medical care? (community organization staff; administrative role)
	We had a client show up for his appointment…he was soaked and it was cold outside, so we were able to thankfully provide him clothes, so he can change into [and] warm up, get some food in his belly, and then he was able to proceed with his appointment. And in that part, that's an example of how we have to provide the flexibility, so we can meet the client where they are, still get the information that we need, and the client can still get the needs and the referrals and the necessities that he needs at that time. (community organization staff; administrative role)
	Once people are released, I think some of their biggest concerns are gonna be financial and transportation, housing. So, if you're able to provide those other things that surround their main health issues, I think people would be more engaged, just because they know that it's like a one-stop shop. You're gonna help me with everything I need. So, I'm going to be successful in following up with care with you once we're done. (county jail personnel; health service provider)
	If we have individuals with specific drug charges or sex crimes, they can have a very hard time finding housing. And then you have those individuals that maybe they're gonna be able to go back to their mom's house or their aunt's house, but it might not be in the best neighborhood or that neighborhood might be a trigger for them as far as staying clean and sober or staying away from risky sexual behavior. So…if they're willing to have the conversation, we can also have those conversations about housing and help them get linked to a case manager [that] can further assist and provide additional referrals. (community organization staff; administrative role)
c) Partnering with community organizations that provide additional social services for individuals upon reentry	You can't try to give them a resource for sexual health, but don't have a resource for housing or don't have a resource for jobs or don't have a resource for whatever need that the client needs. There's a resource for everything. So, you have to be really knowledgeable or [have] the willpower to go out and find the resource. You have to be constantly willing to do the footwork to keep up with the resource. Agencies close, agencies expand, agencies come together. (community organization staff; administrative role)
	[Make] sure that you talk to different organizations in the community because we're not the only ones that have PrEP programs. There are so many resources out there that people just don't know about. Just getting the word out there to everybody and let them know they have options all over the city. And then, they can begin getting PrEP. (community organization staff; administrative role)
	So you have to know where your resources, not only with the organizations, but personally, the people helping these clients need to have personal relationships with people with inside the organization…because I found, when you don't have personal relationships with people inside the organizations, you're just another number or another patient that called in. So having those relationships is really important to navigate all the resources. (community organization staff; administrative role)
d) Overcoming mistrust and stigma	The biggest thing is to actually have somebody who actually is listening to you and not just talking at you. A lot of people are not gonna be open about their lifestyles, they're not gonna tell you that, “Oh, I'm selling myself, I'm prostituting.” They're not gonna be open with that information. So, just listening for those cues to let you know in other ways that that's what they're out here doing, and they need support. We just have to connect with people and get that level of trust because I think people just don't trust healthcare. (community organization staff; administrative role)
	If I approach someone and I'm like warm, welcoming, I ask you about your day. I ask you if there's anything else I can do for you outside of what I'm actually here to ask you for, some people are more inviting and wanting to talk to you and speak with you about things versus if I'm just like, “Hey, I just need these three questions answered and I'm leaving and then you're going back over there and we're just not gonna speak again.” (county jail personnel; health service provider)
	People want you to understand what they're going through, not judge them and give them the resources [and] help that's gonna help them move forward. A lot of times, when you look at somebody sideways, pass judgment, you're not going to be able to help them because they're immediately gonna put their wall up. It's like, “Oh, he think he better than me.” So, I think, really humbling yourself and meeting them where they're at and understanding everybody's not where you're at and if they were where you at, they could possibly be here, but they're not where you're at right now. They're coming to you so they can get past where they're at. (community organization staff; administrative role)
	They may not tell you the first, second or third visit, but that fourth visit when you come back, they're like, “Okay, I'll talk to you, I'll tell you whatever you're asking,” [and] answer your questions. (county jail personnel; health service provider)
	Mostly, my Spanish-speaking patients actually are the ones who are calling me and texting me. I don't speak Spanish. So, of course, we have to text, and I'm using Google translate and going back and forth with you in the middle of the night. And it doesn't matter, if I hear the phone, I'm getting up and I'm gonna respond to you. You want people who are gonna actually be there when the client actually needs them because I've actually had people who converted over to HIV positive because they weren't taking their PrEP correctly. (community organization staff; administrative role)
	Overall, especially with medication adherence, you just have to remember, everybody is gonna have a cell phone with an alarm. That doesn't mean I'm gonna remember to do something at the specific time the alarm goes off or I might just turn it off, because I'm gonna really quickly finish washing dishes and then I completely forget what the alarm was for. (community organization staff; administrative role)
	I just feel like as long as you're showing people that you actually care about their well-being and their health, they tend to gravitate towards you, and they tend to keep up with their appointments and actually do come in. (community organization staff; administrative role)
e) Having relatable providers	If you're able to hire somebody with a history of incarceration, I think that'd be really great, because not only are you providing somebody with a history of incarceration a job, you're also giving this population someone that they can relate to, and it might make it easier for them to open up. (community organization staff; administrative role)
	She [provider colleague] knows all of the logistics and stuff and then I'm very street smart. So, when they ask a question or it's a little thrown off, she might not even catch it sometimes, I kind of know how to respond to them in that area. I'm able to get through some of their walls and to meet them where they're at. I'm able to gain trust sometimes and to reach people that other people can't reach. I've been through some things, you know, I have tattoos, I'm missing a hand, right? So, when I approach people who are down and out or whatever, they look at me and they say, “Oh, she's been through some things herself.” Once you gain the trust, you'll have it forever. (community organization staff; administrative role)
	I think it's really important that our program serves individuals that are ethnic minorities – that's our target population… I think it's really important that your staff has diversity, has people with different genders, ethnicity, sexual orientations, because, me as a female, just the nature of the individuals that are incarcerated, there's more men than women and these men might not be want to talk to a woman or they might not wanna talk to Hispanic women. They might want to talk to somebody that looks completely different. So, I think it's just really important to have a diverse staff and individuals that speak Spanish as well. (community organization staff; administrative role)
f) Communicating candidly and using empowering language	People wanna know exactly – they wanna know the real information. They don't like for things to be sugar-coated. They wanna know exactly what it is. And they want you to be straightforward with them because they read through bull crap. (justice-involved individual)
	I just tell people that if I could take a pill every day and [it] would be 99% effective to not catch cancer, I'm gonna take that pill. It's the same with not catching HIV – 99% chance of not catching HIV if I'm on PrEP. I'm gonna take it. (community organization staff; administrative role)
	Gain frame counseling is essentially when you frame something to a patient, it suggests an intervention and frame it in a positive way, as in, “These are the good things that would happen if you took PrEP,” not, “These are the bad things that will happen if you don't.” So, empowering them in a positive way. (community organization staff; health service provider)
	The way you present it to them, how important it is for their health. You cannot just be, “You should be taking PrEP.” That's not the approach there or you're making them fearful. It's just how you present it, that this is beneficial for them and that they can feel the person genuinely cares for their well-being. (community organization staff; administrative role)
	But I really feel that part of our failure [is] this proper scripting to the person. I did some research on scripting and there is evidence out there that how you present it makes a difference. So, we trained our people. [We] trained our phlebotomy people mostly to try to get them to understand that there's a reason to test. This is not like some stigmatized disease. This is part of a person's healthcare. (county jail personnel; administrative role)
	It seems negative the way it's coming off to inmates and it's received negative. I feel the correctional officers need some education as well. Just a little bit into how to handle some of these situations and how to go about STI testing because it's all sensitive. However they portray it is gonna be how an inmate receives it and comes back to the phlebotomist. If it's like, “Hey, you just need labs,” and then you come over here, “Hey your provider ordered this, we just want to rule it out.” “Okay, cool, go ahead, draw my blood.” But if you're like, “You need an HIV test,” I wouldn't volunteer for that either, especially if you ask me in front of a whole group of people. So, there's a lot of things from different groups that need some work. (county jail personnel; health service provider)
	After we talk about their PrEP compliance, I always say, “And of course we always encourage you to use condoms.” And they sometimes roll their eyes at us, and we laugh and stuff. But ultimately, I always emphasize that this is really about you staying healthy, right? This is what this conversation was about. It just happens to be that you're sexually active. But that's what we emphasize. And then I think without saying that, people are as transparent as they can be, and they are tolerant and non-judgmental. (community organization staff; health service provider)

### Barriers to Linkage to PrEP Care

#### Lack of Capacity for PrEP and HIV Counseling among Medical Providers

Upon arrival at jail, all individuals go through a mandatory health intake. The combination of high individual volume and intensive health needs, further exacerbated by a shortage of jail healthcare providers, places an overwhelming burden on existing providers. Given these limitations, some providers chose to initiate discussions of sexual health only when individuals had a positive STI test in jail, stating that even the presence of other risk factors for adverse sexual health outcomes was insufficient to initiate such a discussion.

Many providers emphasized their limited capacity to add new or different responsibilities to their already heavy workloads. Some stated that for implementation of a PrEP linkage to care program to be effective, it would require its own staffing, given that they are already struggling to fulfill their current responsibilities. Others voiced frustration regarding responsibilities being delegated to them without consideration of their current burden, again voicing their desire for hiring staff dedicated to new activities such as HIV/PrEP.

I feel like if you want [a linkage to care program] to be effective, you're gonna want your own staff to do [it]. Because honestly, everyone here is pretty stretched… If the existing staff does it, they might just do it really quickly. And it's not [going to] be effective. (county jail personnel; administrative role)

#### Limited Knowledge of PrEP among Jail Providers and Justice-Involved Individuals

Both providers and justice-involved individuals reported limited knowledge about HIV and PrEP. Among providers, this lack of knowledge translated to low confidence in providing sexual health education and PrEP care; among justice-involved individuals, it translated to disinterest in HIV preventive care.

One provider described how her professional peers stated that PrEP and sexual health care were outside of their scope of practice. Many jail providers and staff were unfamiliar with where to refer justice-involved individuals for PrEP care outside of the jail.

Who would you tell them to go to for PrEP? We don't even know that… I wouldn't know who to send them to for PrEP right now. (county jail personnel; health service provider)

Similarly, many justice-involved individuals shared that they had only heard about PrEP from commercials or other advertisements. Some providers speculated that some justice-involved individuals may be less interested in PrEP because of misconceptions about PrEP as a treatment for HIV rather than a preventive medication. At the same time, some felt that others accurately understood that PrEP was for people who did not have HIV but underappreciated the value of preventive medications.

#### Stigma Related to Incarceration, Gender and Sexual Minorities, PrEP use, and HIV status

Participants emphasized how stigma at various levels created gaps in linkage to PrEP care. One jail staff member shared that stigma against justice-involved individuals often manifested in the belief among jail officers that incarcerated patients did not deserve free healthcare, yielding a subsequent reluctance among some officers to offer PrEP or ensure individuals received PrEP medications. Another described their opinion that such beliefs among medical staff resulted in disempowering conversations about PrEP with patients in their care, ultimately discouraging justice-involved individuals from pursuing PrEP.

There were both misconceptions and homophobia-related stigma attached to PrEP and HIV among justice-involved individuals, with many believing that PrEP was only appropriate for individuals who identified as sexual minorities or those with HIV. Two justice-involved individuals expressed their discomfort in having discussions about PrEP or HIV in the jail setting out of concern that others might assume that they may be a member of one of these minoritized and stigmatized groups.

It's a stigma when you're in jail about stuff like that. And you really don't want nobody talking to you about it at all, especially if you're not involved in that type of behavior. (justice-involved individual)

One justice-involved individual described how these beliefs could lead to violence towards individuals identified as PrEP users, exacerbating the stigma and further discouraging individuals from being open to PrEP. Relatedly, one jail staff member felt that the mere mention of PrEP could sometimes be misinterpreted as identifying an individual as having HIV or being a sexual minority. On the extreme end, according to one jail provider, PrEP-related stigma led certain justice-involved individuals to decline any discussion whatsoever of their risk factors or need for PrEP.

#### Preexisting Mistrust in the Healthcare and Criminal Justice System among Justice-Involved Individuals

Mistrust in the healthcare system among justice-involved individuals was frequently mentioned as a barrier to linkage to PrEP care. Those working for the jail healthcare system were often assumed to also be connected to the criminal justice system, leading to great mistrust of healthcare workers in general and consequently, a lack of openness among justice-involved individuals with providers. Justice-involved individuals described feeling hesitant to provide accurate contact information to jail personnel, for example, for fear of it being used by the police for tracking purposes. Jail staff noted that this mistrust sometimes complicated future care if they were unable to reach justice-involved individuals after release, for example, to provide test results that had been pending or to facilitate scheduling of a follow-up appointment.

They don't give us good contact information because they're afraid the sheriff will show up at their place and arrest them again. So, it makes it really difficult to do follow-ups because there's that aspect of fear that any information they give may be used against them in the future. (county jail personnel; administrative role)

Jail personnel also reported that many justice-involved individuals were afraid of answering social history questions during health assessments, especially about drug use but even about smoking and alcohol intake, because they were concerned that this information could be used against them by police in the future.

### Facilitators and Strategies to Linkage to PrEP and HIV Care

#### Enhancing Engagement of Justice-Involved Individuals in the Jail Setting Through Digital Media

Several participants stressed the importance of providing education during incarceration about PrEP and HIV. Given that misconceptions and stigma about PrEP use and HIV were common, education was seen as a crucial strategy in motivating individuals to seek HIV preventive care. Although various methods of education were proposed, one innovative approach that had just launched a few months prior to our study was the use of a podcast to provide education on HIV and PrEP to individuals during incarceration. Jail staff commented on the success and popularity of a newly implemented podcast intervention among incarcerated individuals, as it allowed for greater access to education regarding these topics and enabled individuals to engage by submitting questions.

I was surprised by the initial response from the inmates and how engaged they seemed to be. I didn't get to do that podcast for that week, and I came back and checked the little questions where they submitted it and they were like, ‘Where's the podcast? When are you gonna submit them?’ (county jail personnel; health service provider)

The podcasts could also be leveraged to disseminate information to jail staff to debunk misconceptions about PrEP and HIV that lead to stigma, thereby impacting not only the incarcerated population but also correctional officers and other staff who might then be more likely to encourage individuals to seek HIV preventive care.

It's gotten to the point where some of the officers will even be like, “Hey, Texas got the highest number,” because I've said it so many times in front of them and they'll even help me to get people to want to test more. (county jail personnel; health service provider)

#### Addressing Competing Priorities for Justice-Involved Individuals to Make Room for Medical Care upon Community Reentry

Many participants—including justice-involved individuals, jail staff, and community organization staff—spoke about the issue of justice-involved individuals having multiple competing priorities upon reentering the community, including finding housing and employment and fulfilling legal obligations. One care coordinator at the Dallas County Jail suggested the idea of a “one-stop shop,” where individuals could receive medical care and social and legal support in one location, as an easier route to linking an individual to healthcare.

While at the end of the day, the ultimate goal for our program is linkage to care, that might not necessarily be their ultimate goal. So how do we help everybody get what they need? Whether or not that's linkage to care… if you need to do X, Y, Z before medical care, how can we help get that taken care of so that it's easier for you to get linked to medical care? (community organization staff; administrative role)

A community organization staff member also noted that addressing issues like housing could potentially mitigate certain behaviors that can increase exposure to HIV, ultimately leading to better health outcomes in the future.

#### Partnering with Community Organizations That Provide Additional Social Services for Individuals upon Reentry

Although participants stressed the importance of addressing competing priorities prior to linking individuals to healthcare, many recognized that it would take more than one program to provide for the various needs that justice-involved individuals face upon reentry. Thus, many participants stated that it was crucial to know where to find resources in the community so that competing priorities could be adequately met. Several participants noted that this would require a proactive effort by community organizations to coordinate and consolidate resources.

You can't try to give them a resource for sexual health, but don't have a resource for housing or don't have a resource for jobs or don't have a resource for whatever need that the client needs… You have to be constantly willing to do the footwork to keep up with the resource. Agencies close, agencies expand, agencies come together. (community organization staff; administrative role)

In light of the high volume of justice-involved individuals presenting for clinical care, providers also highlighted the importance of partnering with other community organizations specializing in providing PrEP and HIV care in an effort to share the work and increase accessibility to care.

[Make] sure that you talk to different organizations in the community because we're not the only ones that have PrEP programs. There are so many resources out there that people just don't know about. Just getting the word out there to everybody and let them know they have options all over the city. And then, they can begin getting PrEP. (community organization staff; administrative role)

The partnership also meant more than simply having information on an organization and their services. Participants emphasized the difference that having actual connections inside an organization makes in linking an individual to care successfully. Having a personal relationship with someone within the organization allowed providers to advocate for their patients more directly and potentially expedite their care.

#### Overcoming Mistrust and Stigma

Given the combination of mistrust in healthcare and stigma surrounding PrEP and HIV, many participants agreed that effective listening by providers was a key component in building trust and creating space for open and authentic conversations with justice-involved individuals. Many stated that approaching individuals with warmth and genuine care for their well-being helped dissolve the tension and mistrust that justice-involved individuals may initially have, both during and after incarceration.

Humility and empathy were also highlighted as key components of building a trusting relationship, while even slight cues indicating judgment could easily break that bond, potentially stifling an individual's willingness to accept assistance with healthcare or social services. One provider emphasized that building a sincere relationship with justice-involved individuals and breaking down barriers of mistrust took more than an initial encounter and required consistency.

They may not tell you the 1st, 2nd or 3rd visit, but that 4th visit when you come back, they're like, ‘Okay, I'll talk to you, I'll tell you whatever you're asking,’ [and] answer your questions. (county jail personnel; health service provider)

Several community organization members emphasized that care for their clients’ well-being led them to go above and beyond and think outside the box to ensure individuals were receiving the proper attention and care they needed. One community organization member noted the importance of tailoring strategies to accommodate a client's specific needs and lifestyle to help promote adherence to PrEP over time. Many community organization members believed that being authentic in demonstrating one's desire to help went a long way in successfully linking justice-involved individuals to PrEP care.

Overall, especially with medication adherence, you just have to remember, everybody is gonna have a cell phone with an alarm. That doesn't mean I'm gonna remember to do something at the specific time the alarm goes off or I might just turn it off, because I'm gonna really quickly finish washing dishes and then I completely forget what the alarm was for. (community organization staff; administrative role)

#### Having Relatable Providers

Several participants also expressed the importance of having health professionals that justice-involved individuals could relate to in their experiences being homeless, formerly incarcerated, or being in need of other supports. This was key to opening a pathway to gain trust and a greater willingness to engage.

One community organization member talked about bringing her “street smarts,” not necessarily her “book smarts,” to work to build rapport with her justice-involved patients. Many community organization and jail staff agreed that having diverse staff, including those with personal or family experience with incarceration and persons of various ethnicities, sexual orientations, languages, and adverse experiences, was crucial in reaching a broad range of justice-involved individuals.

I've been through some things, you know, I have tattoos, I'm missing a hand, right? So, when I approach people who are down and out or whatever, they look at me and they say, ‘Oh, she's been through some things herself.’ Once you gain the trust, you'll have it forever. (community organization staff; administrative role)

#### Communicating Candidly and Using Empowering Language

All three stakeholder groups emphasized that the way conversations around PrEP and HIV were framed played a significant role in how justice-involved individuals responded. Many shared that using clear and direct language when educating justice-involved individuals about PrEP was preferred by justice-involved individuals and proved to be effective.

One PrEP provider spoke about the concept of “gain frame counseling,” where language surrounding PrEP was centered around benefits of taking PrEP, rather than negative consequences of not taking the medication. More generally, participants stated that using empowering language, rather than language that invokes fear, also built trust between the provider and patient and ultimately resulted in justice-involved individuals being more open to taking PrEP.

The way you present it to them, how important it is for their health. You cannot just be, “You should be taking PrEP.” That's not the approach there or you're making them fearful. It's just how you present it, that this is beneficial for them and that they can feel the person genuinely cares for their well-being. (community organization staff; administrative role)

Language around STI testing in the jail also had the potential to encourage dialogue around PrEP and HIV. One participant shared how phlebotomists on their team were trained to approach testing for STIs as a standard part of healthcare; another participant highlighted that it would be important to train correctional officers in the jail to use sensitive and nonjudgmental language when speaking about testing for STIs.

Ultimately, participants believed that using direct and empowering language is important to show justice-involved individuals that discussions focused on PrEP and HIV care are for the benefit of their well-being and to encourage individuals to take ownership of their health in the best way they can.

## Discussion

Our study highlights critical barriers and facilitators in linking justice-involved individuals to PrEP and HIV care upon community reentry in the context of Dallas, TX, a city in the Southern US that has been identified by the Centers for Disease Control and Prevention as a “hotspot” for HIV infections.^26^ Although similar studies exploring barriers to PrEP care have been conducted in Rhode Island,^24,25^ the Southern US is a key area of interest for improving linkage to PrEP care, as the Southern US accounted for nearly half (49%) of new HIV infections in 2022,^29^ while only 21% of PrEP users were in the South.^30^ Factors such as lack of Medicaid expansion and low rates of health insurance, low health literacy, increased stigma against HIV/PrEP and PrEP, increase in HIV criminalization laws, and low ratios of primary care providers to population have been described as barriers to PrEP linkage to care and uptake that are unique to the Southern US.^31^ One qualitative study examining knowledge and perception of HIV and PrEP among jail detainees has been conducted in Arkansas^21^; however, the perspectives of key stakeholders both in the justice system and in the community have not been surveyed in this region.

Our findings underscore a complex interplay of factors at the individual, dyadic, and structural levels that influence PrEP access and uptake among justice-involved individuals after release that are contextually unique to the Southern US. We found minimal capacity among jail staff and providers to manage PrEP and HIV care, reported lack of PrEP- and HIV-specific knowledge among some jail providers and justice-involved individuals, and multiple forms of stigma related to sexual and gender minorities, incarceration, PrEP, and HIV in both the jail and community.

Previous studies have found that a lack of PrEP knowledge among providers comprised a major barrier to PrEP implementation.^32,33^ We found similar themes among our participants. One participant reported that some providers expressed how their scope of practice was limited to their specialty, which did not include sexual health care. In particular, providers expressed their lack of knowledge regarding PrEP care, not only in providing PrEP care but also in linking patients to providers who prescribed PrEP. In both cases, lack of knowledge and comfort in providing sexual health and PrEP care posed barriers to PrEP linkage and uptake in justice-involved individuals.

Several studies have been conducted on various interventions to address provider confidence and initiation of PrEP discussions as well. Some training programs for providers currently exist, ranging from self-guided training modules for continuing education credits to intensive, in-person trainings spanning several days.^34,35^ Although these programs are not standardized, increased provider knowledge has been linked to greater confidence and willingness to prescribe PrEP and to increased PrEP prescriptions.^33,36,37^ Other studies have shown that automated algorithms can be integrated into electronic health records to prompt providers to initiate PrEP discussions with patients who are likely to benefit from PrEP use, improving efficiency.^38,39^

Multiple layers of stigma—surrounding incarceration, PrEP use, gender and sexual minorities, and HIV status—also presented barriers to engaging in PrEP and HIV care among both providers and justice-involved individuals. One jail staff spoke on how this stigma perpetuated the belief that justice-involved individuals were less deserving of receiving health care or free medications. In addition, justice-involved individuals voiced their hesitations in seeking PrEP care, given the stigma and myths that PrEP was primarily for individuals who were positive for HIV or MSM, echoing the sentiments of justice-involved women in RIDOC.^24^ One jail staff also noted that this stigma could lead to violent situations for those involved in PrEP care, as others not involved with PrEP could resort to violent means to exclude these individuals in an effort to avoid any kind of association with them. Resulting fears, as well as stigmatizing interactions with providers, cause some individuals to avoid utilization of beneficial HIV-related care and lead to negative psychological consequences that ultimately limit access to care.^40^ Additionally, these stigmas are often exacerbated by an entrenched mistrust in the healthcare and criminal justice systems among individuals from racial and ethnic minority groups.

One educational tool that was noted to be effective in spreading awareness of PrEP and dispelling myths and stigma associated with PrEP in our study was a podcast available in the jail. The podcast was initially developed to discuss general sexual health topics; however, with its increasing popularity, jail staff planned to expand the podcast to include additional information including HIV prevention and PrEP. Additionally, after listening to an episode, individuals could submit to health professionals any follow-up questions that emerged to discuss the following week. Podcasts could also be leveraged to disseminate information to jail staff to debunk misconceptions about PrEP and HIV that lead to stigma, thereby impacting not only the incarcerated population but also correctional officers and other staff who might then be more likely to encourage individuals to seek HIV preventive care. Evidence-supported strategies, such as the Finding Respect and Ending Stigma around HIV (FRESH) Workshop, to reduce provider and client stigma have also been developed and piloted, showing high satisfaction and decreased uncertainty about HIV treatment among individuals living with HIV, as well as increased awareness of stigma among providers.^41^ Adapting these strategies for HIV prevention in jail settings and incorporating skills-based training in providing culturally sensitive care around sexual health and substance use disorders may be useful in decreasing stigma-related disengagement among justice-involved individuals and jail providers and rebuilding provider–client trust.

We also found that competing priorities faced by justice-involved individuals upon reentering the community, especially finding housing and employment, and tending to legal requirements, often took precedence over addressing health needs. Previous studies have also reported that healthcare is not a high priority for recently released individuals among other competing needs. Two qualitative studies based in the Rhode Island Department of Corrections that explored barriers to PrEP uptake described how compounding responsibilities and hardships upon reentry led to a deprioritization of PrEP.^24,25^ Similar findings were identified in a qualitative study conducted in Arkansas among jail detainees that reported housing and employment as immediate priorities that were more important than accessing healthcare upon reentry, despite participants reporting a high willingness to use PrEP.^21^

Our results highlight how integrating PrEP services with social support systems, such as through a “one-stop shop” model that combines healthcare with assistance for housing, employment, and other needs may help streamline access to PrEP and reduce barriers to care. Unlocking Doors, a non-profit organization in Dallas, TX, aims to transition individuals reentering society using such a model; their services offer coordinated resources and programs in a combined effort to address the multiple competing needs of an individual.^42^ Medical-legal partnerships also exist across the United States and have been found to have positive health outcomes and increased uptake of social services among varying populations.^43,44^ Several of these partnerships exist in New Haven, CT, working specifically with formerly incarcerated individuals reentering society by placing lawyers and law students in local health clinics so individuals can access both medical and legal services in one location.^45^ Such an approach recognizes the multifaceted challenges faced by those reentering society and aims to provide comprehensive support that addresses medical, socioeconomic, and legal needs.

We found that when justice-involved individuals were asked about their interest in or experience with PrEP, a majority of their responses revolved around barriers that prevented them from initiating or adhering to PrEP. Further studies to assess personal interest in using PrEP for justice-involved individuals and how their degree of motivation to use PrEP may affect the likelihood that they can overcome barriers to access is needed; however, this finding suggests a significant opportunity for initiating PrEP within jails so that key barriers to linkage to care may be minimized before community reentry. In 2019, the Rhode Island Department of Corrections implemented a PrEP initiation program in jails to link incarcerated individuals to PrEP care in the community upon reentry. Though 35% of individuals identified as being at increased risk of HIV acquisition opted to initiate PrEP while incarcerated, less than 3% of individuals were successfully linked to care after release.^19^ Meanwhile, a recent study conducted in Zambia implemented PrEP programs in multiple criminal justice facilities across the country and showed that 93.3% of those reached and found eligible for PrEP initiated PrEP, highlighting the feasibility and potential benefits of starting PrEP within the criminal justice system in some contexts.^46^ The success in Zambia suggests that similar innovative approaches could potentially be effective in the USA, advocating for a reconsideration of current practices and funding allocations, as current USA funding structures and policies, such as a restriction on initiating PrEP in most correctional facilities, present major obstacles. Nevertheless, the findings in Rhode Island also suggest that continued PrEP engagement after release remains an issue potentially even after individuals initiate PrEP within the justice system.

Several considerations in designing and implementing PrEP programs for justice-involved individuals nearing re-entry have previously been described.^18^ These include: identifying points of an individual's involvement in the criminal justice setting that are ideal for PrEP enrollment and provision, establishing protocols and standards of care for PrEP provision or linkage specific to the setting, providing adequate training to providers, and determining the acceptability and feasibility of a program in the setting.^18^ Following such guidelines, while addressing the barriers and facilitators discussed above, could allow for successful implementation of a program that enhances PrEP access and reduces HIV transmission rates, especially in high-risk populations and communities with a particularly high HIV incidence, such as the Southern USA.

Our study design has limitations. This study relied on purposive sampling, particularly with formerly incarcerated individuals attending group meetings held by the HARC and parole orientation. Thus, participants may not represent the views of all justice-involved individuals, jail providers, and community organizations focused on serving incarcerated populations. In addition, we did not implement an eligibility criterion based on time since release from jail or previous incarcerations, which may have affected participants’ recollection of linkage to PrEP services and post-release experiences. While we aimed for diverse perspectives from multiple stakeholders in various roles and organization types, there may still be limitations in capturing a full range of views. For example, individuals not directly involved in PrEP provision or those at different organizational levels may have different insights that were not fully explored. Additionally, given that many of the views expressed were second-hand findings, where individuals reported the views and experiences of others, the findings reported in this study may not accurately represent the views stated. Findings from this study also may not be directly transferable to other regions of the USA with different HIV epidemiology, correctional systems, or community resources, as we aimed to focus our findings in a city with a particularly high burden of both HIV and incarceration. Lastly, given the stigma surrounding topics of HIV, PrEP, sexual and gender minorities, and incarceration, social desirability bias may have prevented participants from fully disclosing their experiences or opinions. Relatedly, while research team members represent some minoritized groups, none have personal experience with the justice system.

## Conclusion

The current study expands on perspectives from professional stakeholders in the jail and community ecosystems, providing details on issues of provider capacity, HIV and PrEP knowledge, competing priorities, and stigma associated with HIV and PrEP, impacting openness to provide and receive HIV and PrEP services. Future research should focus on evaluating the impact of policy changes and funding adjustments on PrEP implementation in jails, as well as novel ways to integrate PrEP services with other social support systems to more effectively address competing priorities faced by justice-involved individuals following community reentry. Additionally, studies should explore how to adapt successful PrEP models in jails from international contexts to local settings, considering the unique challenges and needs of high-burden regions in the USA. By learning from both domestic and international experiences, we can develop more effective strategies for HIV prevention and improve health outcomes for individuals involved in the criminal justice system.
